# Primary Care Triple P for parents of NICU graduates with behavioral problems: a randomized, clinical trial using observations of parent–child interaction

**DOI:** 10.1186/s12887-014-0305-4

**Published:** 2014-12-14

**Authors:** Renske Schappin, Lex Wijnroks, Monica Uniken Venema, Barbara Wijnberg-Williams, Ravian Veenstra, Corine Koopman-Esseboom, Susanne Mulder-De Tollenaer, Ingeborg van der Tweel, Marian Jongmans

**Affiliations:** Department of Medical Psychology and Social Work, Wilhelmina Children’s Hospital, UMC Utrecht, Utrecht, The Netherlands; Department of Child, Family and Education Studies, Faculty of Social and Behavioral Sciences, Utrecht University, Utrecht, The Netherlands; Department of Medical Psychology, Isala Clinics, Zwolle, The Netherlands; Department of Neonatology, Wilhelmina Children’s Hospital, UMC Utrecht, Utrecht, The Netherlands; Department of Neonatology, Isala Clinics, Zwolle, The Netherlands; Julius Center for Health Sciences and Primary Care, UMC Utrecht, Utrecht, The Netherlands

**Keywords:** Primary Care Triple P, Parenting intervention, Preterm birth, Perinatal asphyxia, RCT, Parent–child interaction, Positive parenting skills

## Abstract

**Background:**

Preterm-born or asphyxiated term-born children show more emotional and behavioral problems at preschool age than term-born children without a medical condition. It is uncertain whether parenting intervention programs aimed at the general population, are effective in this specific group. In earlier findings from the present trial, Primary Care Triple P was not effective in reducing parent-reported child behavioral problems. However, parenting programs claim to positively change child behavior through enhancement of the parent–child interaction. Therefore, we investigated whether Primary Care Triple P is effective in improving the quality of parent–child interaction and increasing the application of trained parenting skills in parents of preterm-born or asphyxiated term-born preschoolers with behavioral problems.

**Methods:**

For this pragmatic, open randomized clinical trial, participants were recruited from a cohort of infants admitted to the neonatal intensive care units of two Dutch hospitals. Children aged 2–5 years, with a gestational age <32 weeks and/or birth weight <1500 g and children with a gestational age 37–42 weeks and perinatal asphyxia were included. After screening for a *t*-score ≥60 on the Child Behavior Checklist, children were randomly assigned to Primary Care Triple P (n = 34) or a wait-list control group (n = 33). Trial outcomes were the quality of parent–child interaction and the application of trained parenting skills, both scored from structured observation tasks.

**Results:**

There was no effect of the intervention on either of the observational outcome measures at the 6-month trial endpoint.

**Conclusions:**

Primary Care Triple P, is not effective in improving the quality of parent–child interaction nor does it increase the application of trained parenting skills in parents of preterm-born or asphyxiated term-born children with behavioral problems. Further research should focus on personalized care for these parents, with an emphasis on psychological support to reduce stress and promote self-regulation.

**Trial registration:**

Netherlands National Trial Register NTR2179. Registered 26 January 2010.

**Electronic supplementary material:**

The online version of this article (doi:10.1186/s12887-014-0305-4) contains supplementary material, which is available to authorized users.

## Background

Preterm-born or asphyxiated term-born children who receive neonatal intensive care show more emotional and behavioral problems than term-born children without a medical condition. These children form two major patient groups in the neonatal intensive care unit (NICU), and the prevalence of behavioral problems in these children at (pre)school age is 20%, versus approximately 10% in healthy term-born children [1-3]. Current practice is that parents of preschool-aged NICU graduates are referred to parenting interventions aimed at the general population. However, given the impact of NICU admission on families [4], it is not yet clear whether these generic parenting intervention programs are effective in families with a preterm-born or asphyxiated term-born child.

Transactional theories on the development of behavioral problems in preterm-born children suggest that the interplay between parents’ preexisting personality and family factors, prenatal experiences, and emotional distress during the NICU period, may result in a parenting style that differs from that of parents of healthy termborn children [4]. This parenting style is characterized by overprotection and inconsistent discipline [5], and may be due to both parenting stress and the perception of parents of their preterm-born preschooler as still being vulnerable [6]. In combination with the neurological predisposition to emotional and behavioral problems, these overprotective and inconsistent parenting practices may negatively impact the behavior of the child [7,8]. Furthermore, parenting stress in itself is a strong predictor of emotional and behavioral problems in preterm-born children (Schappin R, Wijnroks L, Uniken Venema M, Jongmans M: Predictors of change in behavioral problems over a 1-year period in preterm born preschoolers, submitted).

Existing interventions for preterm-born and asphyxiated term-born children aimed at improving developmental outcomes exclusively take place during the neonatal period and the first year of life [9,10]. Examples of these interventions are the Infant Behavioral Assessment and Intervention Program (IBAIP) and the Maternal Infant Transaction Program (MITP) [11,12]. IBAIP guides parents in supporting their infant’s self-regulatory competence, by sensitizing them to their infant’s responses during interactions with the environment. MITP tries to enable parents to appreciate the unique characteristics of their child, to sensitize them for their infant’s cues, and teaches parents how to respond appropriately to these cues. IBAIP and MITP are two of the very few interventions in the first year of life that have been shown to be effective beyond this first year. Children who received IBAIP have a lower percentage of performance IQ scores below 85, and better scores on subtasks of intelligence and motor tests at age 5, compared to children who did not receive IBAIP [13]. At 2 years, children who received MITP have better communication skills and their parents have less parenting stress compared to children who did not receive MITP [14,15].

Although the effects of these early interventions are positive, they do not seem to have an effect on emotional and behavioral problems [14,16]. Furthermore, these interventions are developed specifically for the neonatal and infancy period and can therefore not be used to reduce child problem behavior during the preschool period or beyond. Since problem behavior usually surfaces around two years of age in preterm-born children [17], there is a need for parenting interventions for parents of NICU graduates at preschool age. Therefore, in these families, we investigated the effectiveness of a widely-used brief parenting intervention named Primary Care Triple P. Triple P is a stepped-care system of parenting interventions that aims to reduce child problem behavior by improving the competences of parents in terms of their parenting behavior, in parents of children between 0 and 12 years old [18,19]. A brief version of Triple P was chosen because it seemed to fit the problems reported by parents during regular clinical follow-up. Furthermore, at the time our trial was designed, several studies had demonstrated the effectiveness of Primary Care Triple P in non-clinical populations [20,21].

The present study on observational outcomes of Primary Care Triple P is an extension of the findings from our trial on parent- and teacher-reported outcomes of Primary Care Triple P [19]. Surprisingly, the parent- and teacher-reported outcomes of our trial indicated that Primary Care Triple P was not effective in reducing emotional and behavioral problems in preterm-born children or term-born children with perinatal asphyxia. However, the working mechanism behind Triple P suggests that child problem behavior is reduced through enhancement of the quality of the parent–child interaction, by improving parental competences [18]. Therefore, even when parent-reported emotional and behavioral problems in children do not decrease, Primary Care Triple P may still increase parenting competences, which could provide evidence for the effectiveness of part of the Triple P system.

The most objective method to assess whether parenting behaviors have changed is by structured observations of parent–child dyads. Two recent meta-analyses of Triple P have both included studies that used observations of parenting behaviors to assess the effect of Triple P [22,23]. In all of the included studies the measurement used to assess observations was the Family Observation Schedule Revised, a tool that is part of the Triple P program and consists of scoring the incidence of predominantly negative parent and child behaviors [24]. The first recent meta-analysis was conducted in 2012 and included 23 published studies of any level of Triple P in comparison to a control condition [22]. Seven studies that used independent observations of parent and child behavior were identified. Of these seven studies, only two reported significant effects in favor of Triple P on at least one subscale of the observational measure. In 2014, another meta-analysis on Triple P was published that analyzed 101 studies on any version of Triple P, including unpublished studies [23]. Short-term effects in favor of Triple P were found on independent observations of child behavior for every level of Triple P except for Primary Care Triple P. No significant effects were found for short-term effects on observations of parent behavior, nor on long-term effects on observations of both child and parent behavior. In both meta-analyses, parent-reported and observational outcomes on negative child behavior differed remarkably, with larger effects in favor of Triple P on parent-reported child behavior compared to observations of child behavior. These results emphasize the importance of including observational measures in trials of parenting interventions.

With regard to our previous findings that Primary Care Triple P was not effective in reducing child problem behaviors [19], in the current study we investigated whether Primary Care Triple P would nonetheless improve parental competences in parents of preterm-born or asphyxiated term-born preschoolers with emotional and behavioral problems. When parental competences are indeed improved, this could provide evidence for the effectiveness of the working mechanism of Triple P: to change child behavior through parenting behavior. This is the first study that conducted independent observational measures to assess the effect of Primary Care Triple P, and the first study of any level of Triple P that assessed the observation of specific parenting skills that were trained during the Triple P intervention.

## Methods

### Study design

Participants were originally recruited for a pragmatic, open randomized clinical trial that investigated the effectiveness of a Primary Care Triple P in terms of parent- and teacher-reported child behavioral problems, in preterm-born and asphyxiated term-born preschoolers [19]. The structured observations of parent–child dyads were part of this trial and were conducted in both the intervention group and the wait-list control group. Two Dutch medical centers with a NICU participated in the study: the University Medical Center Utrecht/Wilhelmina Children’s Hospital (Utrecht) and the Isala Clinics (Zwolle). Approval for the study was obtained from the institutional review boards of both centers (the ‘Medisch Ethische Toetsingscommissie’ of the University Medical Center Utrecht and the ‘Medisch Ethische Toetsingscommissie’ of the Isala Clinics).

The sample size calculation was based on the parent-reported questionnaire outcome of the original study, the Child Behavior Check List (CBCL). Assuming no decline in the CBCL *t*-score of the wait-list control group, the sample size was based on the possibility to detect a difference of 5 points on the CBCL total problem *t*-score between the intervention and control group at the 6-month primary outcome measurement. Because a decrease in behavioral problems in the intervention group compared to the control group was expected, a one-sided 5% significance level was chosen. With a power of 80%, an attrition rate of 10%, and a standard deviation of 7.1 based on preliminary findings, we calculated that we needed a minimum sample size of 32 children per treatment arm.

### Participants

Participants were recruited by mail from a cohort of infants born between September 2004 and October 2007 and subsequently admitted to the NICUs of the two participating centers. Children of 2 to 5 years of age, born with gestational ages <32 weeks or birth weights <1500 g were eligible for the screening phase of this study, together with children born at a gestational age of 37–42 weeks showing clinical signs of perinatal asphyxia (Apgar score <5 at 5 minutes, umbilical cord arterial pH <7.10, prolonged resuscitation, and acidosis). We excluded children with cognitive and/or motor impairments (defined as a developmental quotient <70 and/or a Gross Motor Function Classification System score >3 [25]), children with parents who did not speak Dutch, and children from families that had received a parenting intervention in the last 6 months.

Eligible children and their families who provided written consent were screened for children’s emotional and behavioral problems. Children whose parents reported a *t*-score ≥60 on the internal, external, or total problem scale of the Child Behavior Checklist (CBCL) were eligible for randomization [26]. Parents’ informed consent was obtained separately for the screening and randomized phase of the study.

### Intervention

Primary Care Triple P is a brief parenting intervention that consists of 4 sessions involving active skills training for parents of children with mild to moderate emotional or behavioral problems. Sessions took place in one of the two participating hospitals once a week, with a break of 3 weeks before the fourth session, and both parents were encouraged to attend sessions. The main objective of Triple P is to reduce child problem behavior by improving parent competence and self-reliance in terms of parenting [18].

The Primary Care Triple P training was provided by one experienced social worker, two registered healthcare psychologists, and two registered clinical psychologists. They received three-and-a-half-days of training in Primary Care and Standard Triple P and passed an individual examination and accreditation test to become licensed Triple P practitioners. Peer supervision between these professionals and the first author conducted at least once a month assured adherence to the intervention protocol.

Parents all completed four sessions. There were 21 parents who attended sessions together, 12 mothers and 1 father came alone. During the first 6 months of the study, the intervention group did not receive any intervention other than Primary Care Triple P. At 6 months after the start of the study, intervention group children and their parents requiring additional support received further treatment from their assigned Triple P professional or were referred to other health care providers when necessary. We ensured that the control group children and their parents did not receive any intervention until 6 months after the start of the study. At this time, control group children and their parents could opt to receive Primary Care Triple P or another type of psychological treatment suited to their problems, or to be referred to other health care professionals.

### Observational measures

Children and parents participated in observations at baseline, immediately after completion of the intervention (approximately 2 months), 6 months, and 12 months after the start of the intervention, all in one of the two participating medical centers. The time-point of 6 months was the time of the primary outcome of this trial. At 12 months, a follow-up measure was conducted. Immediately after their last Primary Care Triple P session, the intervention group parents also completed the Client Satisfaction Questionnaire (CSQ; parent evaluation of the program) in the hospital [27]. Neonatal variables were obtained from the child’s medical records. Family background variables were assessed at baseline.

The observation task used in this study was the Three Boxes Task, adapted from the National Institute of Child Health and Human Development (NICHD) Early Child Care Research Network protocol [28]. Although this task has slightly different versions for different ages of the child, for comparability we chose to use the 36 months version throughout our study. In this task, the room is set up with an adult’s and a children’s table, a soft rug on the floor and three numbered boxes of toys. The parent who spends the most time with the child, was asked to participate in the task. Upon entering the room, the parent was given the following instructions: “This task will take about 15 minutes. Please help your child play with the toys in the three boxes in the way you would at home if you were able to spend some free time alone with your child. Let your child start with the first box and finish with the third box. During the third box, your child should play alone with the toys and you can complete a questionnaire at the adult’s table. After the third box, the room should be cleaned up. You can change boxes or start cleaning up when you hear a knock on the mirror or door. There is a bowl with boxes of Smarties and raisins on the children’s table, which you cannot remove. Your child is allowed to pick a sweet after the room is cleaned up”. Short instructions were also written down on a small index card for the parent. Answers to parent’s questions about the task were kept intentionally vague (“Do whatever you might do at home”), to ensure naturally occurring diversity in parenting behaviors.

The first box contained toys for imaginary play: two fake mobile phones, a girl baby doll with clothes, a sword, a firemen’s helmet, a child-size Zorro cape, and an adult-size golden glitter vest. The second box contained drawing materials: blank paper, colored crayons with a sharpener, two pencils with sharpener and shape templates of geometrical shapes, animals, and vehicles. The shape templates enabled the parent to teach their child how to use them. The third box contained toys for the child to play on its own: Duplo building blocks, a cash register, and an excavator. To ensure that parents would invest in their children playing alone, they were given a questionnaire on parenting that needed their full attention. Parents and children were given five minutes to play with each box.

The Three Boxes Task was assessed with two different scoring systems. The first scoring system measures the quality of parent–child interaction and is used with the Three Boxes Task in the NICHD Early Child Care Research Network [28]. As for the observation task itself, we chose to use only the 36 months version of the scoring system throughout the study. The qualitative scoring system has five parent scales: (1) supportive presence, positive regard and emotional support to the child; (2) respect for child autonomy, recognizing and respecting the validity of the child’s individuality, motives, and perspectives; (3) stimulation of cognitive development, fostering the child’s cognitive and mental development; (4) hostility, expressions of anger, discounting or rejecting the child; and (5) confidence, the belief in the ability to work successfully with the child and that the child will behave appropriately. There are also four child scales: (1) enthusiasm, acting with vigor, confidence, and eagerness; (2) negativity, showing anger, dislike, or hostility towards the parent; (3) persistence, the extent to which the child was involved with the toys; and (4) affection towards parent, substantial periods of positive regard and happy feelings towards the parent. Finally, the qualitative scoring system has one dyadic scale: felt security, the availability of mutuality of emotions between child and parent, and how secure the child feels with the parent. All scales are scored on a 7-point range from 1 (very low) to 7 (very high). Furthermore, there is an extended description for behaviors that fall under each point of each scale. For example, parents score 4 points (moderate) on hostility when they show “Several instances of hostile or rejecting behaviors. Two or more of these events are reliably clear to observers, but expressions are brief and do not set the tone of parent’s interactions immediately following the episodes” [29].

The second scoring system that was used to assess the Three Boxes Task was a quantitative system. The system was based on the 17 core parenting strategies that are part of every level of the Triple P intervention. Because not all parenting strategies could be used by parents in the Three Boxes Task, or did not have a distinctive capacity due to the nature of the task, 6 parenting strategies were excluded from the scoring system (spending quality time, talking with children, providing engaging activities, setting a good example, using behavior charts, and establishing ground rules). The remaining 11 parenting skills that were included in the quantitative scoring system were: showing affection; using non-descriptive praise (parents should use descriptive praise instead); using descriptive praise; using incidental teaching; using ask, say, do; using directed discussion for rule breaking; using planned ignoring for minor problem behavior; giving clear, calm instructions; backing up instructions with logical consequences; using quiet time for misbehavior; and using time-out for serious misbehavior [27]. The quantitative system consisted of counting the instances that a parent showed one of the parenting skills.

Both scoring systems were scored from standardized videotapes by an independent observer who was blind to both the intervention-status and the time of measurement of the parent–child dyads. The videos were standardized so that they contained exactly the first 3 minutes of play with each box and the first 1 minute of cleaning up. The same observer, an experienced child psychologist, scored all videos. To assess reliability, approximately 10% (n = 17) of the videos were scored by a second observer, a junior child psychologist. Both observers were trained by the second author of this article (LW). Agreement within 2 points on the qualitative rating scales ranged from Cohen’s kappa = .52 (moderate; *P* < .001) for the scale supportive presence, to Cohen’s kappa = 1.00 (perfect; *P* < .001) for the scales hostility, enthusiasm, and negativity [30]. On the quantitative scales, the parenting skills quiet time and time-out were not used by parents, logical consequences was used only three times. For the remaining parenting skills, agreement within 2 points ranged from Cohen’s kappa = .57 (moderate; *P* < .001) for the parenting skill incidental teaching, to Cohen’s kappa = 1.00 (perfect; *P* < .001) for the scales showing affection, directed discussion, and planned ignoring.

### Procedures

Random assignment of families to either the intervention or the control group was stratified for each center. Due to the nature of the intervention, when twins were included both siblings received the same treatment. Open allocation of children to either the intervention or control group at a ratio of 1:1 was performed by the first author, in the order of receiving the informed consent forms, according to computer-generated random permuted blocks of 6 [31]. After the trial’s primary endpoint at 6 months, children and their parents in both the intervention and the control group received appropriate (additional) psychological treatment when needed. Therefore, at the 12-month follow-up, groups were analyzed according to the intention-to-treat principle, but allocation to treatment was no longer random.

Linear mixed models were used to estimate the effects of Primary Care Triple P on the observational outcomes. Linear mixed models have the advantage over repeated measures analysis of variance that they are able to handle missing data and uneven spacing between time-points. Within linear mixed models, changes from baseline to successive time-points in the intervention group are compared to changes in the same time-period for the control group. In this study, differences between the intervention and control group, development of parent–child interaction in the randomized phase (baseline to 6 months – 3 time-points) and non-randomized phase (6 months to 12 months – 2 time-points) of the study, and the interaction between group and time were investigated. Group, time-point, and the interaction between group and time-point were included as fixed effects in the model. Intercept was included as a random effect. Because there we only 2 to 3 time-points in the models, a random slope was not estimated. Including the random intercept in the model enables variation in individual levels of the outcome variable. Covariance structures are specified in the results table, and were first selected based on model fit (likelihood ratio test) and second on model simplicity. The repeated measures covariance structure defines the relation between variances and covariances in the model for the repeated effect (time). For example, the assumption that variances are the same at each time-point would lead to a different covariance structure than the assumption that variances are different at each time-point. Because we included only one random effect in our models, the covariance structure for the random effect was scaled identity. Model fit was assessed using IBM SPSS version 20 [32], with REML estimation. Missing values were not imputed. All available data were used, with analyses based on the intention-to-treat principle.

## Results

### Participants

**Figure 1:**
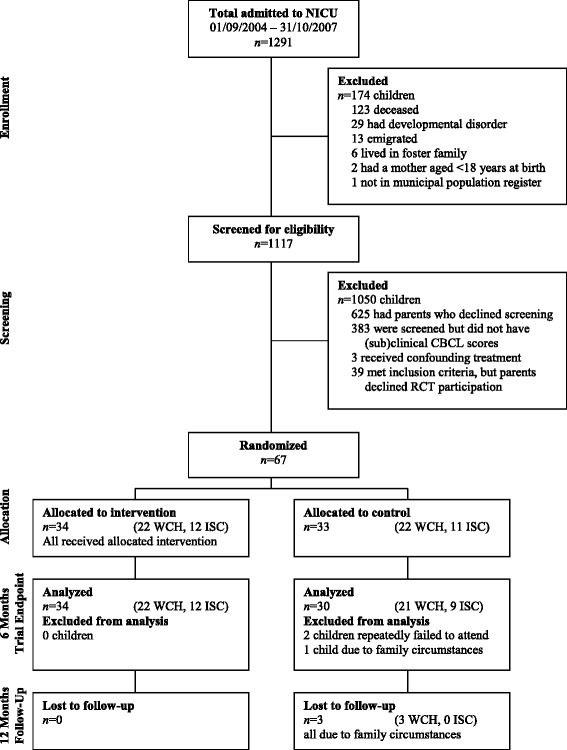
**Participant flow through screening, trial, and follow-up.** Abbreviations: WCH, Wilhelmina Children’s Hospital; ISC, Isala Clinics.

Between May 2009 and March 2010, we recruited parents of 2- to 5-year-old children who had been admitted to the NICUs of the Wilhelmina Children’s Hospital and the Isala Clinics. Because of infant mortality or known ineligibility for screening, 174 children were excluded beforehand. The 1117 remaining children were all approached for screening, and 492 children’s parents consented. Of these 492 children, 106 met inclusion criteria for randomization, and 67 children’s parents consented to participate in the randomized trial. The children participating in the randomized clinical trial were randomly allocated to the intervention (n = 34) or control group (n = 33). See Figure 1 for participant flow.

During the first 6 months of the trial, 3 children dropped out of the control group: 1 child’s parents were not able to take leave from work, and 2 children’s parents repeatedly failed to complete questionnaires or participate in the Three Boxes Task. By the 12-month follow-up, 3 more children had dropped out, respectively due to emigration, a mother’s second complicated pregnancy, and severe illness in the family. The Three Boxes Task was completed 247 times in total. Recording failed in 12 (4.9%) of these observations, so 235 videos could be scored. Failed recordings are spread evenly across the intervention and control groups, but not across time-points: baseline, 1 intervention and 1 control recording failed; directly after the intervention, 1 intervention recording failed; primary outcome, 2 intervention and 4 control recordings failed; and follow-up, 2 intervention and 1 control recording failed.

In the control group, 17 children and their parents received an intervention after 6 months; in the intervention group, 6 children and their parents received an additional intervention. In both groups, depending on the child’s or parents’ problems, interventions could range from a one-hour session with a psychologist, to 4 sessions of Primary Care Triple P, to referral to a child psychiatrist.

Baseline characteristics of children and parents are presented in Table 1. Although many children were part of a twin or triplet, there was only one twin of which both children were included in the study (intervention group). There were no significant differences in demographic and neonatal characteristics between the total cohort and the RCT participants. We did not test for baseline differences between the intervention and control group. Since participants were allocated at random to study groups, all differences between groups are coincidental.

**Table 1:** **Baseline characteristics of children and parents participating in the intervention and control group** ^**a**^

	**Intervention (n = 34)**	**Control (n = 33)**
**Child characteristics**		
Age, mean (SD), mo	45.6 (10.0)	43.6 (10.7)
Males	16 (47%)	24 (73%)
Children part of a twin/triplet	5 (15%)	4 (12%)
BW, mean (SD), g	1477.1 (849.3)	1626.7 (876.3)
GA, mean (SD), wk	30.5 (4.2)	30.8 (4.5)
Abnormal cerebral ultrasound		
IVH grade 1-2	31 (91%)	31 (94%)
IVH grade 3-4	3 (9%)	2 (6%)
Perinatal asphyxia (term-born)	3 (9%)	5 (15%)
NICU stay, mean (SD), d	21.4 (20.1)	18.6 (16.2)
**Family characteristics**		
Maternal age, mean (SD), y	34.1 (5.5)	32.2 (5.2)
Maternal ethnicity		
European	33 (97%)	33 (100%)
North-African	1 (3%)	0
Firstborn child	26 (77%)	27 (82%)
Maternal education, mean (SD), y	14.5 (2.1)	14.7 (2.0)
Paternal education, mean (SD), y	14.2 (2.8)	14.8 (2.4)
Family situation		
Nuclear family	32 (94%)	30 (91%)
Stepfamily	1 (3%)	0
Single parent family	1 (3%)	3 (9%)
Mother participated in observation	115 (96%)	123 (92%)

*Abbreviations:* *BW* birth weight, *GA* gestational age, *IVH* intraventricular hemorrhage.

^a^Data are presented as number (percentage) unless otherwise specified.

### 6-Month trial endpoint outcomes

The analysis of the qualitative scoring system outcomes at the 6-month trial endpoint is presented in Table 2; the analysis of the quantitative scoring system outcomes is presented in Table 3. Means and standard deviations for each time-point are presented in Additional file 1. The analyses include three time-points: the baseline measure, the measure directly after completion of the intervention, and the 6-month trial endpoint measure. On the qualitative scoring system, there was a significant mean difference in parental confidence between the Primary Care Triple P intervention group and the wait-list control group, with more confidence in the intervention group. However, in the absence of an interaction effect between the intervention and time of measurement, this difference cannot be ascribed to Primary Care Triple P. There was significant variation in the intercept of supportive presence, parents’ respect for child autonomy, and parents’ hostility. This indicates that there was significant variation among parent–child dyads in their levels of quality of parent–child interaction on these three scales.

**Table 2:** **Estimated fixed and random effects for qualitative observation outcomes from baseline to 6-month trial endpoint**

		**Fixed effects**						**Random effects**	
**Outcome**	**n**	**Intervention**	***P***	**Time**	***P***	**Intervention × Time**	***P***	**Intercept**	***P***
**Parent**									
Supportive presence^a^	66	0.50 (0.55)	.371	−0.10 (0.08)	.210	0.13 (0.11)	.226	0.97 (0.48)	.043
Respect child autonomy^a^	66	−0.03 (0.64)	.960	0.16 (0.08)	.057	0.06 (0.11)	.625	1.94 (0.72)	.007
Cognitive development^a^	66	0.23 (0.56)	.675	−0.13 (0.08)	.117	0.18 (0.12)	.129	0.12 (0.40)	.755
Hostility^b^	66	0.46 (0.48)	.343	−0.01 (0.06)	.891	−0.07 (0.09)	.443	0.66 (0.28)	.021
Confidence^a^	66	1.46 (0.56)	.010	0.05 (0.08)	.542	−0.08 (0.11)	.466	0.51 (0.47)	.284
**Child**									
Enthusiasm^a^	66	0.53 (0.53)	.319	0.07 (0.07)	.337	−0.02 (0.10)	.839	0.82 (0.45)	.064
Negativity^a^	66	0.03 (0.49)	.946	−0.05 (0.07)	.452	0.03 (0.09)	.768	0.67 (0.36)	.064
Persistence^a^	66	0.20 (0.65)	.753	0.14 (0.09)	.149	0.11 (0.13)	.394	0.86 (0.60)	.152
Affection^a^	66	0.59 (0.47)	.206	−0.03 (0.07)	.703	0.02 (0.10)	.833	0.12 (0.29)	.669
**Dyadic**									
Felt security^a^	66	0.74 (0.55)	.178	−0.02 (0.08)	.774	0.06 (0.11)	.562	0.51 (0.44)	.244

Note. Because the random effect has only one level, the covariance structure is scaled identity.

^a^Repeated measures covariance structure is scaled identity.

^b^Repeated measures covariance structure is diagonal.

**Table 3:** **Estimated fixed and random effects for quantitative observation outcomes from baseline to 6-month trial endpoint**

		**Fixed effects**						**Random effects**	
**Outcome**	**n**	**Intervention**	***P***	**Time**	***P***	**Intervention × Time**	***P***	**Intercept**	***P***
Showing affection^a^	66	0.05 (0.14)	.716	−0.004 (0.02)	.843	0.001 (0.03)	.972	0.07 (93.45)	.999
Non-descriptive praise^b^	66	−0.71 (0.70)	.313	−0.07 (0.10)	.464	0.19 (0.14)	.165	1.17 (0.71)	.097
Descriptive praise^b^	66	−0.16 (0.29)	.568	−0.004 (0.04)	.915	0.05 (0.05)	.368	0.34 (0.13)	.008
Incidental teaching^c^	66	0.09 (0.24)	.701	−0.02 (0.04)	.523	0.01 (0.05)	.857	0.27 (291.82)	.999
Ask, say, do^d^	66	0.48 (0.22)	.036	0.02 (0.03)	.398	−0.10 (0.04)	.020	0.08 (0.05)	.103
Directed discussion^d^	66	0.17 (0.25)	.497	−0.003 (0.03)	.934	−0.01 (0.04)	.808	0.10 (0.04)	.012
Planned ignoring^d^	66	0.13 (0.13)	.308	−0.02 (0.02)	.369	−0.02 (0.02)	.513	0.003 (0.01)	.834
Clear, calm instructions^b^	66	0.41 (0.31)	.193	−0.02 (0.05)	.602	−0.01 (0.06)	.816	0.17 (0.14)	.219

Note. Because the random effect has only one level, the covariance structure is scaled identity.

^a^Repeated measures covariance structure is toeplitz.

^b^Repeated measures covariance structure is scaled identity.

^c^Repeated measures covariance structure is compound symmetry correlation metric.

^d^Repeated measures covariance structure is diagonal.

For the quantitative scoring system, there was a significant difference between the Primary Care Triple P intervention group and the wait-list control group on the parenting skill ‘using ask, say, do’, with more use of ‘ask, say, do’ in the Triple P group. There was also a significant interaction effect between intervention and time of measurement on the skill ‘using ask, say, do’. The interaction effect indicates that the intervention group showed a decrease in the use of ‘ask, say, do’ from baseline to the 6-month trial endpoint, whilst the control group showed an increase in the use of ‘ask, say, do’ from baseline to the 6-month trial endpoint. There were two significant random intercepts for the quantitative scoring system; individual parents differed in their levels of descriptive praise and directed discussion.

### 12-Month follow-up outcomes

The analysis of the 12-month follow-up outcomes for the qualitative scoring system is presented in Table 4; the analysis of the quantitative scoring system is presented in Table 5. These analyses include two time-points: the 6-month trial endpoint and the 12-month follow-up outcome measure. There was a significant mean difference between the Primary Care Triple P group and the intervention group on the scales supportive presence, stimulation of cognitive development, and the dyadic scale felt security, in favor of the Triple P group. For two scales, there were also significant interaction effects between the intervention and time of measurement. Stimulation of cognitive development and dyadic felt security decreased in the Triple P intervention group from 6 to 12 months, whilst there was an increase of parental stimulation of cognitive development and dyadic felt security in the control group from 6 to 12 months. Besides these interaction effects, there was significant variation in the intercept of supportive presence, parental hostility, and child persistence, indicating that there was significant variation among parent–child dyads in their levels of quality of parent–child interaction on these three scales.

**Table 4:** **Estimated fixed and random effects for qualitative observation outcomes from 6-month trial endpoint to 12-month follow-up**

		**Fixed effects**						**Random effects**	
**Outcome**	**n**	**Intervention**	***P***	**Time**	***P***	**Intervention × Time**	***P***	**Intercept**	***P***
**Parent**									
Supportive presence^a^	58	3.53 (1.38)	.013	0.001 (0.11)	.993	−0.29 (0.15)	.053	1.32 (0.65)	.044
Respect child autonomy^a^	58	2.18 (1.83)	.238	0.12 (0.14)	.409	−0.23 (0.19)	.249	1.75 (1.17)	.137
Cognitive development^b^	58	4.21 (1.67)	.015	0.15 (0.14)	.277	−0.39 (0.18)	.034	0.39 (3.54)	.912
Hostility^a^	58	−0.08 (1.25)	.947	0.002 (0.10)	.981	0.01 (0.13)	.940	1.21 (0.55)	.028
Confidence^a^	58	1.19 (1.46)	.419	−0.05 (0.11)	.659	−0.09 (0.15)	.574	1.04 (0.71)	.139
**Child**									
Enthusiasm^c^	58	0.15 (1.45)	.919	−0.005 (0.11)	.963	−0.04 (0.14)	.788	0.91 (0.56)	.102
Negativity^c^	58	0.34 (1.41)	.808	−0.15 (0.10)	.149	−0.01 (0.14)	.934	0.04 (0.35)	.899
Persistence^a^	58	1.75 (1.69)	.303	−0.04 (0.13)	.745	−0.18 (0.18)	.325	1.95 (0.99)	.047
Affection^c^	58	2.48 (1.38)	.077	0.07 (0.10)	.511	−0.24 (0.13)	.078	0.52 (0.43)	.225
**Dyadic**									
Felt security^c^	58	3.46 (1.55)	.030	0.11 (0.11)	.340	−0.33 (0.15)	.034	1.00 (0.60)	.096

Note. Because the random effect has only one level, the covariance structure is scaled identity.

^a^Repeated measures covariance structure is scaled identity.

^b^Repeated measures covariance structure is compound symmetry heterogeneous.

^c^Repeated measures covariance structure is diagonal.

**Table 5:** **Estimated fixed and random effects for quantitative observation outcomes from 6-month trial endpoint to 12-month follow-up**

		**Fixed effects**						**Random effects**	
**Outcome**	**n**	**Intervention**	***P***	**Time**	***P***	**Intervention × Time**	***P***	**Intercept**	***P***
Showing affection^a^	59	−0.02 (0.41)	.967	−0.03 (0.03)	.328	0.01 (0.04)	.885	0.02 (0.04)	.627
Non-descriptive praise^a^	59	−1.37 (2.23)	.543	0.06 (0.18)	.738	0.25 (0.24)	.288	2.88 (1.68)	.087
Descriptive praise^a^	59	0.02 (0.95)	.980	−0.02 (0.08)	.779	0.02 (0.10)	.845	0.19 (0.28)	.490
Incidental teaching^a^	59	0.24 (0.72)	.740	0.09 (0.06)	.140	0.004 (0.08)	.961	0.22 (0.18)	.203
Ask, say, do^c^	59	0.57 (0.50)	.264	0.03 (0.04)	.393	−0.07 (0.05)	.178	0.13 (433.84)	1.000
Directed discussion^b^	59	0.14 (0.39)	.723	−0.02 (0.03)	.470	0.01 (0.04)	.753	0.21 (0.06)	<.001
Planned ignoring^c^	59	0.04 (0.24)	.859	−0.001 (0.02)	.949	0.002 (0.03)	.938	0.02 (28.08)	.999
Clear, calm instructions^a^	59	1.36 (0.80)	.095	0.02 (0.06)	.698	−0.15 (0.08)	.087	0.66 (0.26)	.012

Note. Because the random effect has only one level, the covariance structure is scaled identity.

^a^Repeated measures covariance structure is scaled identity.

^b^Repeated measures covariance structure is scaled identity diagonal.

^c^Repeated measures covariance structure is compound symmetry correlation metric.

For the quantitative scoring system, there were only two significant random intercepts; individual parents differed in their levels of directed discussion and giving clear, calm instructions.

## Discussion

This study showed that Primary Care Triple P was not effective in improving the quality of parenting behaviors and the application of trained parenting skills in parents of preschool-aged preterm-born children or asphyxiated term-born children. There was no significant difference in favor of Primary Care Triple P between the intervention group and the control group at the 6-month trial endpoint. At the 12-month follow-up, most measures showed no changes in parenting behavior. However, when changes were present positive parenting behaviors decreased in the Primary Care Triple P group, whilst they increased or remained stable in the control group. The increase in positive parenting behaviors in the control group could be due to the seventeen children and their parents that received an intervention after six months of waiting. The decline of positive parenting behaviors in the intervention group is more difficult to explain, but may be due to receiving less research-related attention during the follow-up period. Our present findings are in line with our earlier findings on parent-reported child behavioral problems in the same group of families [19]. We found no positive effect of Primary Care Triple P on parent-reported child beha vioral problems in this study, although behavioral problems decreased in both the intervention and control group. Interestingly, we do not see this decrease in our independent observations of child behavior. This could be an indication that it is merely the perception of parents that has changed during our study, and not actual child behavior.

In our earlier publication on the parent-reported child behavior outcomes of Primary Care Triple P, we discussed that our lack of positive results could be due to the specific characteristics of our population or a general lack of effectiveness of the Triple P program [19]. We investigated the effectiveness of Primary Care Triple P specifically in NICU graduates because in practice, parents of preschool-aged NICU graduates are referred to parenting interventions aimed at the general population. Taken all together, our findings suggest that a generic parenting program like Primary Care Triple P is not suitable for parents of NICU graduates, given their specific problems. Another issue is that a recent meta-analysis found that Primary Care Triple P was more effective in studies involving Triple P developers [22]. In particular, developer-led studies of Triple P consistently show larger effects on child behavior than studies by independent researchers, who generally report smaller to non-existent effects of Triple P. However, another meta-analysis did not find this difference [23]. Nonetheless, there are some doubts about the generalizability and transferability of the Triple P program.

Our assumption is that the lack of effectiveness of Triple P in our study may be due to unmet needs of the parents of our specific NICU graduate population. Parents of preterm-born and term-born asphyxiated infants experience stress and anxiety, and sometimes even depression during their child’s admission to the NICU [33]. Although preterm or problematic birth may give rise to parental distress, the extent of the distress does not seem to be exclusively dependent on the severity of the preterm infant’s illness. Other factors, such as the sex of the parent or the mother’s age at birth also influence the level of parental distress [34]. Parental distress may impact parenting behavior, leading to less sensitive, intrusive, and more negative parenting [35]. However, although the impact of these parenting behaviors may be negative for term-born children, some of these parenting styles may be adaptive for preterm-born children. For example, maternal directive parenting seems to improve executive functioning in preterm-born, one year old infants (Van de Weijer-Bergsma E, Wijnroks L, Van Haastert IC, Boom J, Jongmans MJ: Maternal interactive styles and individual differences in developmental trajectories of attention and executive functioning in infants born preterm, submitted). Nonetheless, negative and maladaptive parenting behaviors may influence the behavior of the child, leading to more emotional and behavioral problems [36].

Notwithstanding, the path from preterm or asphyxiated birth to behavioral problems may be even more complex than described above. Children may be diversely susceptible to parenting behaviors. Infant’s temperamental difficulty and low sustained attention may interact with parenting behavior to influence children’s emotional and behavioral outcome [37]. Besides parenting, there may be a direct link between the neurological vulnerability of preterm birth and behavioral problems [8,38]. Furthermore, preterm birth is associated to socio-economic circumstances, and these adverse circumstances may also influence behavioral problems [38,39].

Looking at the problems of parents of preterm-born and term-born asphyxiated children, their needs may be more specific than the needs that are addressed in the Triple P program. Although the general aim of Triple P to enhance the knowledge, skills and confidence of parents is probably appropriate for all parents, it may not be sufficient for parents of NICU graduates. These parents could be more in need for an intervention focused on the reduction of stress and promotion of self-regulation.

There are several limitations of our study. Only few fathers participated in our observations. Therefore, our outcomes can best be interpreted as maternal outcomes, and we have little information on the impact of Primary Care Triple P on father’s behavior. Furthermore, we included children with gestational ages <32 weeks and term-born children with perinatal asphyxia. This was because of their twofold risk for emotional and behavioral problems. Due to the nature of our sample, our results are not generalizable to late preterm-born children or healthy term-born children.

The major strength of our study is that it is the first randomized clinical trial that used observational measures of parent–child interaction that were independent of the Triple P program to investigate the effectiveness of Primary Care Triple P. Furthermore, it is the first randomized trial that used observational measures of parent’s use of trained Triple P parenting skills to investigate any level of Triple P.

## Conclusions

In this randomized clinical trial, Primary Care Triple P was not effective in improving the quality of parent–child interaction nor did it increase the application of trained parenting skills in parents of preterm-born or asphyxiated term-born children with emotional and behavioral problems. Further research should focus on personalized care for these parents, with an emphasis on psychological support to reduce stress and promote self-regulation.

## Additional file

Additional file 1:
**Means and standard deviations of qualitative and quantitative observation scores at each time-point.** Description: This file provides two tables, one for the qualitative and one for the quantitative observation scores. For both scoring systems the means and standard deviations at each time-point of the study are given.
