# Supplementary material for: Primary Care Triple P for parents of NICU graduates with behavioral problems: a randomized, clinical trial using observations of parent–child interaction
Source: BMC Pediatr. 2014 Dec 14;14:305. doi: 10.1186/s12887-014-0305-4 (PMC4273431; doi:10.1186/s12887-014-0305-4)
Supplement: Additional file 1: — Means and standard deviations of qualitative and quantitative observation scores at each time-point. Description: This file provides two tables, one for the qualitative and one for the quantitative observation scores. For both scoring systems the means and standard deviations at each time-point of the study are given. [file 12887_2014_305_MOESM1_ESM.pdf]

**Table 1 – Mean and standard deviation of qualitative observation scores at each time-point**

| | Baseline ($n_i$ = 33 / $n_c$ = 31) | | 2 months ($n_i$ = 32 / $n_c$ = 30) | | 6 months ($n_i$ = 30 / $n_c$ = 22) | | 12 months ($n_i$ = 28 / $n_c$ = 25) | |
|---|---|---|---|---|---|---|---|---|
| | Intervention Mean (SD) | Control Mean (SD) | Intervention Mean (SD) | Control Mean (SD) | Intervention Mean (SD) | Control Mean (SD) | Intervention Mean (SD) | Control Mean (SD) |
| Supportive presence | 12.52 (2.12) | 12.00 (1.90) | 12.50 (2.50) | 11.57 (2.36) | 12.87 (2.15) | 11.27 (2.39) | 11.43 (1.73) | 11.20 (2.12) |
| Respect child autonomy | 10.85 (2.14) | 10.87 (2.90) | 11.81 (2.75) | 11.80 (2.11) | 12.47 (2.79) | 11.82 (2.68) | 11.86 (2.55) | 12.24 (2.67) |
| Cognitive development | 7.24 (2.18) | 6.97 (2.21) | 7.13 (2.76) | 6.20 (1.88) | 7.77 (2.37) | 6.14 (1.73) | 6.61 (2.06) | 6.84 (2.17) |
| Hostility | 4.64 (1.83) | 4.16 (1.86) | 4.16 (1.44) | 4.13 (1.41) | 4.17 (1.90) | 4.14 (1.93) | 4.29 (2.09) | 4.24 (1.76) |
| Confidence | 13.73 (2.54) | 12.32 (2.52) | 13.53 (1.92) | 12.27 (1.91) | 13.53 (2.29) | 12.95 (2.40) | 12.82 (1.47) | 12.68 (2.29) |
| Enthusiasm | 12.97 (2.14) | 12.52 (2.38) | 13.41 (2.06) | 12.63 (1.88) | 13.20 (2.16) | 13.23 (2.29) | 12.96 (1.53) | 13.28 (1.79) |
| Negativity | 4.79 (2.01) | 4.71 (2.34) | 4.63 (1.66) | 4.53 (1.72) | 4.63 (2.20) | 4.36 (1.79) | 3.86 (1.11) | 3.64 (1.32) |
| Persistence | 10.85 (2.35) | 10.71 (2.84) | 12.06 (2.84) | 11.07 (1.89) | 12.53 (3.16) | 11.95 (2.26) | 11.39 (2.11) | 11.72 (2.44) |
| Affection | 12.00 (1.50) | 11.39 (2.03) | 11.69 (2.24) | 11.17 (1.26) | 12.13 (1.96) | 11.27 (2.16) | 11.29 (1.05) | 11.56 (1.83) |
| Felt security | 11.67 (2.31) | 10.94 (2.42) | 11.78 (2.21) | 10.80 (1.54) | 12.03 (2.04) | 10.77 (2.76) | 10.93 (1.30) | 11.20 (2.06) |

**Table 2 – Mean and standard deviation of quantitative observation scores at each time-point**

| | Baseline ($n_i$ =33 / $n_c$ =31) | | 2 months ($n_i$ =32 / $n_c$ =30) | | 6 months ($n_i$ =30 / $n_c$ =22) | | 12 months ($n_i$ =29 / $n_c$ =25) | |
|---|---|---|---|---|---|---|---|---|
| | Intervention Mean (SD) | Control Mean (SD) | Intervention Mean (SD) | Control Mean (SD) | Intervention Mean (SD) | Control Mean (SD) | Intervention Mean (SD) | Control Mean (SD) |
| Showing affection | 0.30 (0.59) | 0.26 (0.58) | 0.25 (0.57) | 0.17 (0.46) | 0.30 (0.54) | 0.27 (0.70) | 0.17 (0.60) | 0.12 (0.33) |
| Non-descriptive praise | 2.85 (2.41) | 3.61 (2.69) | 4.09 (3.01) | 3.53 (2.71) | 3.20 (3.09) | 2.77 (2.84) | 4.62 (3.80) | 3.20 (3.33) |
| Descriptive praise | 0.61 (0.93) | 0.77 (1.18) | 0.84 (1.05) | 0.77 (1.38) | 0.90 (1.24) | 0.73 (1.20) | 0.86 (1.75) | 0.64 (0.81) |
| Incidental teaching | 0.73 (1.15) | 0.61 (0.99) | 0.44 (0.76) | 0.43 (0.90) | 0.77 (1.10) | 0.50 (0.91) | 1.17 (1.00) | 0.92 (1.12) |
| Ask, say, do | 0.79 (1.08) | 0.26 (0.68) | 0.28 (0.58) | 0.50 (0.68) | 0.40 (0.72) | 0.32 (0.57) | 0.21 (0.56) | 0.48 (0.71) |
| Directed discussion | 0.39 (1.27) | 0.16 (0.58) | 0.22 (0.49) | 0.17 (0.46) | 0.37 (0.89) | 0.14 (0.35) | 0.31 (0.71) | 0.04 (0.20) |
| Planned ignoring | 0.30 (0.59) | 0.16 (0.45) | 0.22 (0.49) | 0.23 (0.68) | 0.10 (0.31) | 0.05 (0.21) | 0.10 (0.41) | 0.04 (0.20) |
| Clear, calm instructions | 2.06 (1.27) | 1.65 (1.20) | 1.91 (1.28) | 1.63 (1.16) | 1.77 (1.43) | 1.36 (1.26) | 1.21 (1.21) | 1.52 (1.19) |
